# Amoxicillin removal by babassu-derived activated carbon under ultrasonic irradiation: adsorption performance and tree-based machine learning modeling

**DOI:** 10.1007/s11356-026-38361-z

**Published:** 2026-10-29

**Authors:** Luiz Eduardo Carvalho Medeiros, Rossean Golin, Jéssica Aparecida Ribeiro Ambrosio, Andreza Ribeiro Simioni, Amanda Alcaide Francisco Fukumoto, Eduardo Beraldo de Morais

**Affiliations:** 1 Postgraduate Program in Water Resources, Federal University of Mato Grosso, Cuiabá, MT Brazil; 2 Department of Sanitary and Environmental Engineering, Federal University of Mato Grosso, Cuiabá, MT Brazil; 3 Research and Development Institute, Vale Do Paraíba University, São José Dos Campos, SP Brazil

**Keywords:** Emerging contaminants, Adsorption, Isotherms, Kinetic, Adsorption mechanism, Extra Tree model

## Abstract

The contamination of aquatic environments by antibiotics has become a major environmental concern due to their persistence and contribution to antimicrobial resistance. In this study, activated carbon derived from babassu endocarp (BAC) was evaluated for the removal of amoxicillin (AMX) from aqueous solutions under ultrasonic irradiation. BAC exhibited a heterogeneous porous structure with a specific surface area of 623.38 m^2^ g^−1^. Adsorption equilibrium was achieved within approximately 10 min under ultrasonic irradiation. This equilibrium time is shorter than equilibrium times reported for many activated carbons used for AMX removal. The Freundlich model provided the best fit to the equilibrium data, while the Langmuir model predicted a maximum adsorption capacity of 185.57 mg g^−1^. Thermodynamic analysis indicated that the adsorption process was spontaneous and endothermic. BAC also maintained satisfactory adsorption performance after four regeneration cycles. In addition, tree-based machine learning algorithms, including Decision Tree (DT), Random Forest (RF), and Extra Trees (ET), were applied to predict adsorption capacity, with the ET model achieving the best predictive performance (*R*^2^ = 0.983) in the test dataset. Feature importance analysis identified the initial AMX concentration as the most influential operational variable. The adsorption mechanism was proposed to involve pore filling, hydrogen bonding, π–π interactions, and electrostatic attractions. Overall, the results demonstrate that BAC is a promising low-cost adsorbent for rapid AMX removal and highlight the applicability of machine-learning approaches for adsorption process prediction.

**Supplementary Information:**

The online version contains supplementary material available at https://doi.org/10.1007/s11356-026-38361-z.

## Introduction

The contamination of water resources has emerged as a critical environmental challenge, fueled by the rising prevalence of emerging contaminants (ECs)—such as pharmaceuticals, personal care products, pesticides, microplastics, and nanomaterials—in surface and groundwater systems (Cangola et al. 2024; Yu et al. 2024; Zhang et al. 2024). These substances threaten ecological stability and human health by disrupting endocrine systems, fostering antibiotic resistance, and compromising aquatic biodiversity (Li et al. 2024a; Manjarrés-López et al. 2025; Mofijur et al. 2024; Nishmitha et al. 2025). Their ubiquity is largely linked to diverse sources, including municipal and hospital effluents, industrial discharges, agricultural runoff, and landfill leachates (Das and Raj 2025; Dubey et al. 2023; Li et al. 2024b).

Among ECs, antibiotics are of particular concern due to their extensive application in human and veterinary medicine, aquaculture, and livestock production. A significant fraction of administered antibiotics is excreted in pharmacologically active forms, leading to their persistence in aquatic environments and the continuous exposure of microbial populations (Nishmitha et al. 2025). This chronic presence accelerates the dissemination of antibiotic-resistant bacteria and resistance genes, potentially disrupting microbial communities and essential biogeochemical cycles (Fatimazahra et al. 2023; Kong et al. 2024; Liu et al. 2025; Medha et al. 2025). Furthermore, antibiotics may induce detrimental sublethal effects in non-target organisms, such as algae, invertebrates, and fish (Islam and Midya 2023; Li et al. 2024b; Yun et al. 2023).

Amoxicillin (AMX), a prominent β-lactam antibiotic, is frequently detected in water bodies due to its high global consumption and substantial excretion rate—often exceeding 80% in its unchanged form (Singh et al. 2024; Souza et al. 2023). Its continuous discharge, coupled with its limited removal efficiency in conventional wastewater treatment plants, contributes to its environmental persistence. This scenario poses significant ecological risks and underscores the urgent need for efficient, scalable, and cost-effective removal strategies.

Several treatment technologies, including advanced oxidation processes (AOPs), membrane filtration, and biological methods, have been investigated for antibiotic removal (Aryee et al. 2022; Zhu et al. 2024). However, these approaches present significant technical and economic limitations: AOPs often involve high energy demands and the potential formation of toxic degradation byproducts; membrane systems suffer from fouling and high operational costs; and biological treatments frequently result in incomplete degradation (Chen et al. 2022; Li et al. 2024a; Mirizadeh et al. 2024; Mofijur et al. 2024; Nishmitha et al. 2025).

Adsorption has emerged as a premier technique due to its operational simplicity, versatility, and exceptional ability to sequester contaminants even at trace concentrations (Aryee et al. 2022; Nascimento et al. 2024). While various adsorbents—including metal–organic frameworks (MOFs), clays, nanoparticles, and biochar—have shown promising performance, activated carbon (AC) stands out as a leading material (Ahmadi et al. 2024; Haskan and Oymak 2024; Pooresmaeil et al. 2025; Souza et al. 2023; Seymen et al. 2025). The prominence of AC is attributed to its high specific surface area, well-developed pore structure, and diverse surface functional groups, complemented by its regeneration potential and proven scalability for industrial applications (Belaissa et al. 2022; Martins et al. 2025; Yusop et al. 2025a).

AC can be sustainably synthesized from agro-industrial and extractive residues, aligning with the core principles of the circular economy. Various biomass-derived ACs have demonstrated exceptional performance in AMX sequestration, including those produced from coffee waste, palm trunk, murici seeds, and the shells of walnut, pistachio, and pine nut (Laksaci et al. 2023; Martins et al. 2025; Seymen et al. 2025; Yusop et al. 2025a). Among these, the endocarp of babassu (*Attalea speciosa*) has attracted attention as a promising precursor. Its high carbon content, inherent structural rigidity, and widespread availability through traditional extractivist practices in Brazil—particularly in the North and Northeast regions—make it an ideal candidate for the production of high-performance activated carbon with significant socio-environmental value (Ferreira et al. 2023).

Adsorption efficiency is governed by several operational parameters, including pH, temperature, initial concentration, adsorbent dosage, and contact time (Elnazer et al. 2024). Among these, contact time is particularly important for the practical implementation of adsorption processes, as conventional systems are often limited by slow mass transfer and prolonged equilibrium times (Chakinala and Gogate 2024). Consequently, various strategies have been investigated to facilitate mass transfer and reduce the time required to reach adsorption equilibrium. In particular, ultrasonic irradiation has attracted increasing attention because acoustic cavitation creates localized hydrodynamic effects that have been reported to reduce diffusion limitations and improve mass transfer during adsorption processes (Bisaria et al. 2022; Das et al. 2023). However, the magnitude of these effects depends strongly on the characteristics of the adsorbent, the adsorbate, and the operating conditions (Han et al. 2025). Accordingly, evaluating different adsorbent materials under ultrasonic irradiation remains important for advancing the understanding of adsorption behavior under these operating conditions.

In parallel, machine learning (ML) techniques have emerged as robust tools for modeling and optimizing adsorption processes, particularly in systems governed by complex, nonlinear interactions (Nascimento et al. 2024). ML approaches can significantly reduce experimental workload, identify latent patterns, and support strategic decision-making by predicting system performance across diverse operational scenarios (Santos et al. 2025). Specifically, tree-based algorithms—such as Decision Tree, Random Forest, and Extremely Randomized Trees—have demonstrated superior predictive accuracy and robustness in complex adsorption matrices (Seymen et al. 2025).

Although adsorption under ultrasonic irradiation and machine learning have individually shown considerable potential for improving water treatment processes, their combined application for AMX removal using biomass-derived activated carbon remains largely unexplored. Therefore, this study investigates the removal of amoxicillin by babassu-derived activated carbon under ultrasonic irradiation. In addition to evaluating the adsorption behavior through kinetic, equilibrium, thermodynamic, regeneration, and mechanistic analyses, tree-based machine learning models were developed based on the experimental dataset to predict adsorption capacity and identify the relative importance of the operational variables.

## Material and methods

### Chemicals and materials

**Fig. 1:**
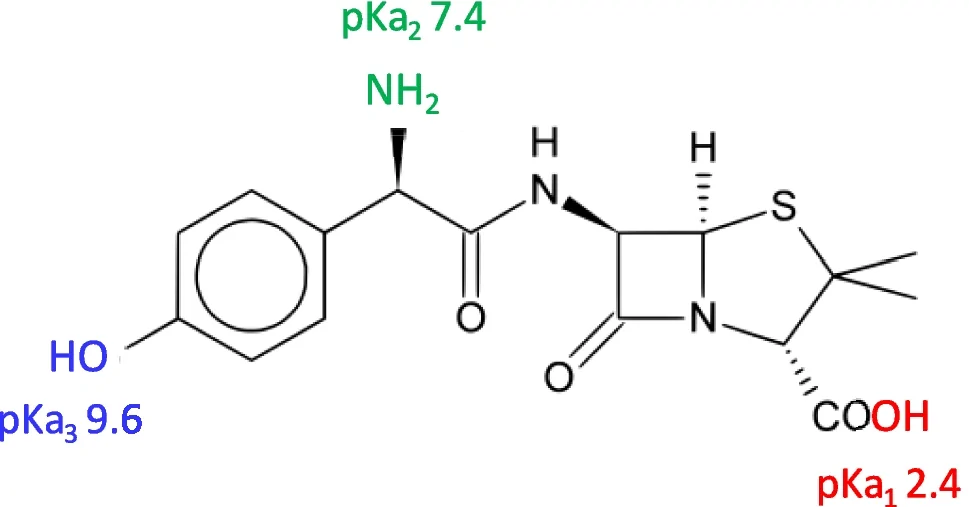
Chemical structure of AMX

Amoxicillin (AMX) in powder form was purchased from Unichem Farmacêutica (Brazil). The chemical structure of AMX is presented in Fig. 1. A stock solution was prepared by dissolving a precise amount of the antibiotic in distilled water, followed by subsequent dilutions to the required concentrations. Solutions of HCl (0.1 M) and NaOH (0.1 M) were used for pH adjustment. The babassu coconut activated carbon (BAC) was provided by Tobasa Bioindustrial (Brazil). All reagents used in this study were of analytical grade and used without further purification.

### Characterization of adsorbent

The BAC was subjected to several analyses to evaluate its morphological and physicochemical properties. The surface morphology was examined using scanning electron microscopy (SEM, Zeiss EVO-MA10) operated at an accelerating voltage of 20 kV. Fourier transform infrared spectroscopy (FTIR) was employed to identify the functional groups on the BAC surface and to investigate their interactions with AMX; the spectra were recorded using a Shimadzu IRAffinity-1 spectrometer. The surface area and pore structure of the adsorbent were evaluated via nitrogen adsorption/desorption at − 196 °C using a surface area analyzer (JWGB BK400). The Brunauer–Emmett–Teller (BET) and Barrett − Joyner − Halenda (BJH) methods were used to determine the specific surface area and pore size distribution, respectively (Rodrigues et al. 2020). Additionally, the point of zero charge (pH_PZC_) was determined using the pH drift method (Laksaci et al. 2023).

### Ultrasonic-assisted adsorption studies

Ultrasound-assisted adsorption experiments were performed using a thermostatic water bath system coupled with a probe-type ultrasonic processor (QSonica Q55, QSonica LLC, USA) equipped with a 6.4 mm titanium tip operating at a fixed frequency of 20 kHz and a nominal maximum power output of 55 W. The sonication was carried out in continuous mode with the amplitude set at 10%. The detailed configuration of the ultrasonic apparatus is presented in Fig. S1 (Supplementary Material). The adsorption performance was evaluated under varying experimental conditions, including pH (2–10), BAC dosage (0.2–1.0 g L^−1^), initial AMX concentration (10–200 mg L^−1^), contact time (2–18 min), and temperature (10, 25, 40, and 50 °C). For comparison, selected adsorption experiments were also conducted under static conditions, without ultrasonic irradiation, using otherwise identical experimental conditions. All adsorption experiments were performed in triplicate (*n* = 3), and the reported values represent the mean ± standard deviation.

After each adsorption run, the BAC was removed from the medium by filtration through 0.45 µm membrane, and the residual concentration of AMX was determined by UV–Vis spectrophotometry at the maximum absorption wavelength (λ = 228 nm). The adsorption capacity at time *t* (*q*_t_, mg g^−1^) was calculated according to Eq. (1):

1 $${q}_{t}=\frac{({C}_{0}-{C}_{t})}{B}$$

where *C*_0_ (mg L^−1^) represents the initial AMX concentration, *C*_t_ (mg L^−1^) is the AMX concentration at time *t* after adsorption, and *B* (g L^−1^) is the BAC dosage in the solution.

### Assessment of AMX stability under ultrasonic irradiation

To evaluate the possible degradation of AMX caused by ultrasound, control experiments were performed in the absence of BAC. AMX solutions (20 mg L^−1^) were subjected to sonication for 30 min under the same operating conditions employed in the adsorption experiments. UV–Vis spectra were recorded at 0, 10, 20, and 30 min of sonication.

### Kinetic, equilibrium and thermodynamic studies

Adsorption kinetic experiments were conducted at various initial AMX concentrations (20, 50, 100 mg L^−1^), with contact times ranging from 2 to 18 min, using a BAC dosage of 0.2 g L^−1^ at 25 °C. The experimental data were fitted to three kinetic models: pseudo-first-order, pseudo-second-order, and intraparticle diffusion.

Equilibrium studies were performed using initial AMX concentrations from 10 to 200 mg L^−1^, maintaining a constant BAC dosage (0.2 g L^−1^) and temperature (25 °C). The resulting isotherms were analyzed using the Langmuir, Freundlich, and Dubinin–Radushkevich models. Furthermore, a thermodynamic evaluation was conducted by calculating the changes in Gibbs free energy (Δ*G*), enthalpy (Δ*H*), and entropy (Δ*S*) to elucidate the nature of the adsorption process. The mathematical expressions for all kinetic, isotherm, and thermodynamic models are provided in the Supplementary Material (Tables S1 and S2).

### Machine learning modelling

Three tree-based machine learning (ML) algorithms were employed to model the adsorption behavior of AMX onto BAC: Decision Tree (DT), Random Forest (RF), and Extremely Randomized Trees (ET).

DT is an interpretable algorithm that recursively partitions the dataset based on feature thresholds, generating a tree-like structure of decision rules. In regression tasks, predictions correspond to the mean value of samples within each terminal node (Wang et al. 2024). RF is an ensemble-learning algorithm that reduces overfitting by constructing multiple decision trees through bootstrap resampling and aggregating their predictions, thereby improving model robustness and generalization capability (BinMakhashen et al. 2024). ET, also known as Extra Trees, introduces a higher degree of randomness by randomly selecting both features and split thresholds during tree construction. This increased diversity often reduces model variance and improves predictive performance for complex nonlinear datasets (Zhang et al. 2026).

All ML models were developed in Python using the scikit-learn library. The machine learning dataset consisted of 48 experimental observations obtained from the adsorption experiments performed in this study. The input variables were the initial solution pH, initial AMX concentration, BAC dosage, temperature, and contact time, while the adsorption capacity (*q*, mg g^−1^) was used as the output variable. The dataset was randomly divided into training (70%) and testing (30%) sets (Santos et al. 2025). To reduce the risk of overfitting during hyperparameter optimization, five-fold cross-validation was employed within the training dataset, while the independent test set was reserved exclusively for the final evaluation of model performance. The hyperparameter search space explored during GridSearchCV and the corresponding optimal hyperparameter values for each model are provided in Table S3 (Supplementary Material).

Model performance was evaluated using the coefficient of determination (*R*^2^), root mean squared error (RMSE), and mean absolute error (MAE). Parity plots were generated to visually assess the correlation between experimental and predicted *q* (mg g^−1^) values. Additionally, feature importance analysis was performed based on the reduction in variance (impurity) to quantify the relative contribution of each input parameter to the model output (Zhang et al. 2020).

### Regeneration and reuse of activated carbon

The regeneration and reusability of BAC were evaluated through successive adsorption–desorption cycles. After each adsorption run, the spent adsorbent was separated and subjected to regeneration using a 0.1 M NaOH solution as the eluent to promote AMX desorption. The regeneration process was intensified by ultrasound, where the BAC was suspended in the NaOH solution and subjected to ultrasonic irradiation for 30 min.

Subsequently, the material was exhaustively washed with distilled water to remove residual NaOH and desorbed species until a neutral pH was achieved. The BAC was then dried in a convection oven at 105 °C for 24 h. The regenerated material was reused in subsequent cycles under identical experimental conditions (initial AMX concentration: 20 mg L^−1^; BAC dosage: 1.0 g L^−1^; contact time: 10 min; temperature: 25 °C). These adsorption–desorption cycles were repeated to evaluate the reusability of the adsorbent over successive adsorption–desorption cycles.

## Results and discussion

### BAC characterization

**Fig. 2:**
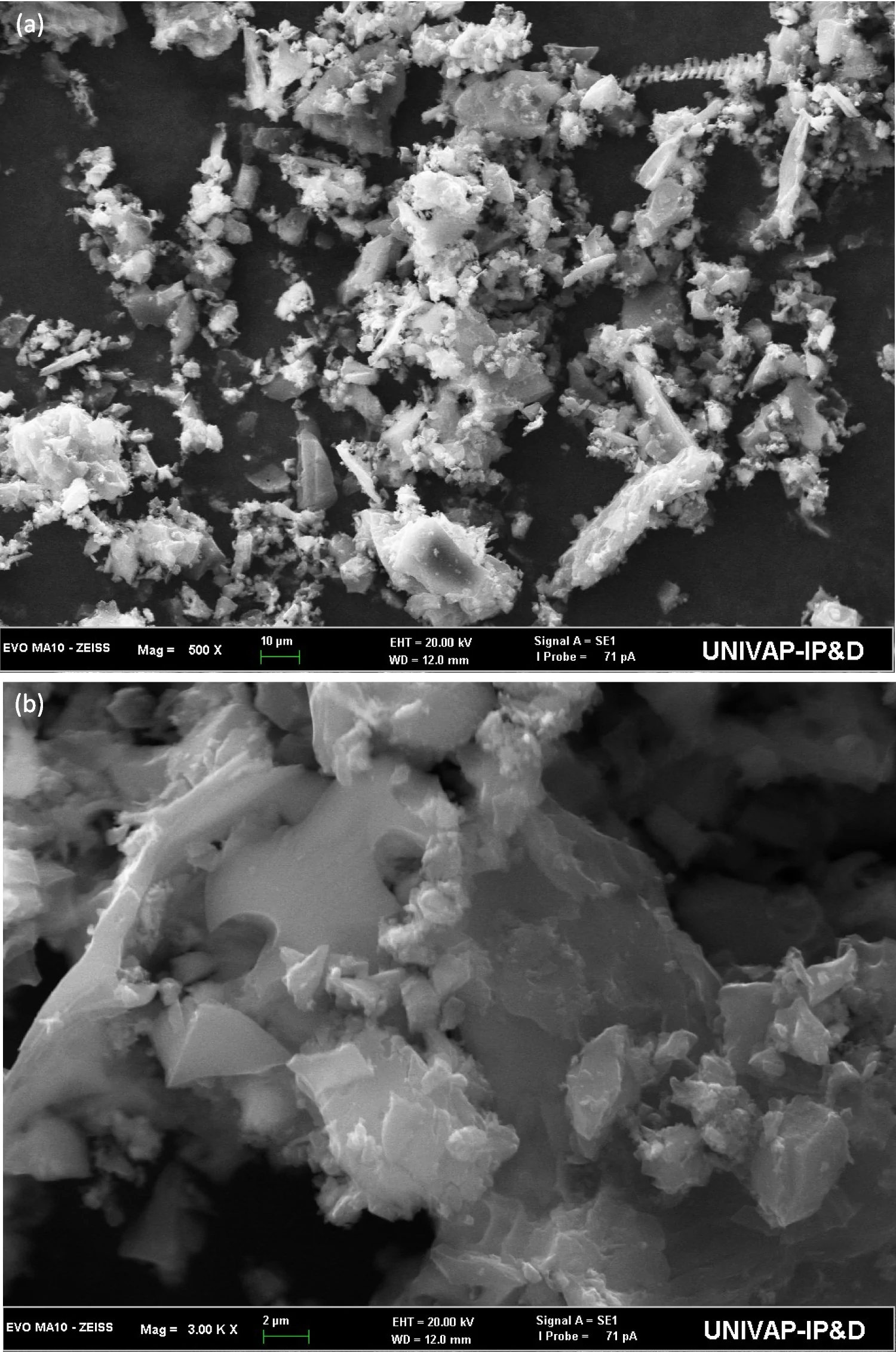
SEM images of BAC at 10 µm scale (**a**) and 2 µm scale (**b**)

SEM micrographs revealed that BAC consists of heterogeneous particles with diverse morphologies and dimensions (Fig. 2). The surface exhibits pronounced irregularities, including cracks, rough textures, and protrusions—features typically associated with the chemical and thermal activation of lignocellulosic precursors (Dolfini et al. 2024). This complex topography is beneficial for adsorption, as it provides a large number of potential contact sites for AMX molecules.

**Fig. 3:**
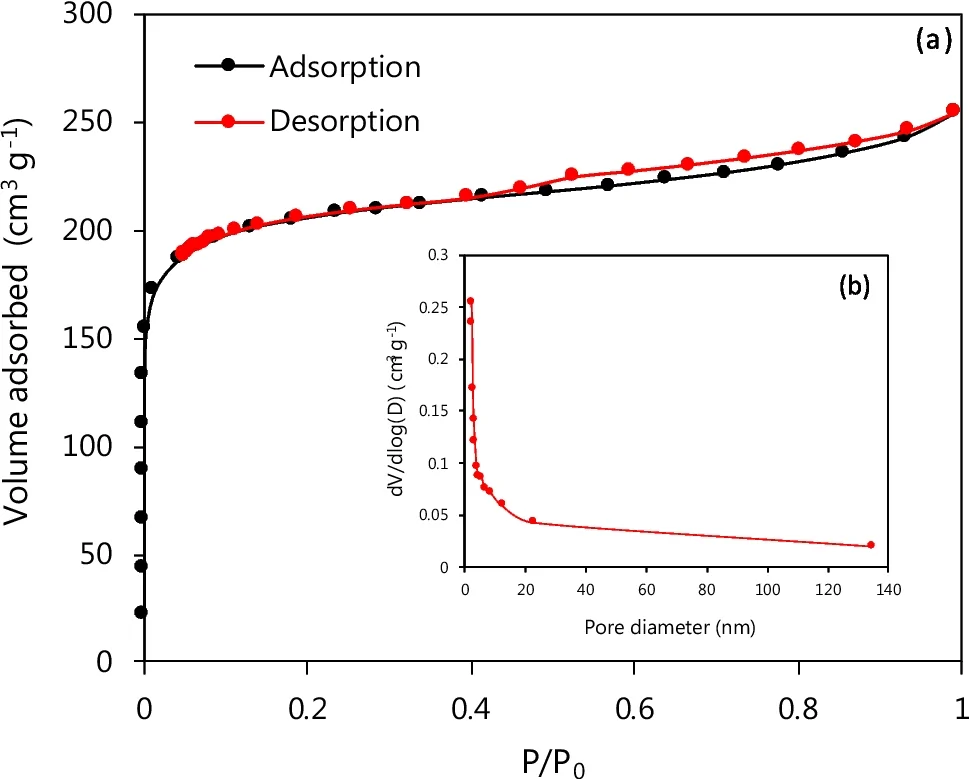
N_2_ adsorption and desorption isotherm (**a**) and pore size distribution curve for BAC (**b**)

While SEM provides qualitative information regarding surface morphology, quantitative characterization of the pore structure was obtained from N_2_ adsorption–desorption analyses (Fig. 3). According to the IUPAC classification, the isotherm exhibits combined characteristics of type I and type IV profiles. The sharp uptake of N_2_ at low relative pressures (P/P_0_ < 0.1) indicates the presence of micropores, whereas the hysteresis loop observed at intermediate relative pressures (P/P_0_ = 0.45–0.95) confirms the existence of mesoporosity (Shi et al. 2023). BAC exhibited a specific surface area of 623.38 m^2^ g^−1^, a total pore volume of 0.393 cm^3^ g^−1^, and an average pore diameter of 2.52 nm. These textural characteristics likely favored the diffusion and adsorption of AMX molecules within the porous structure of BAC.

**Fig. 4:**
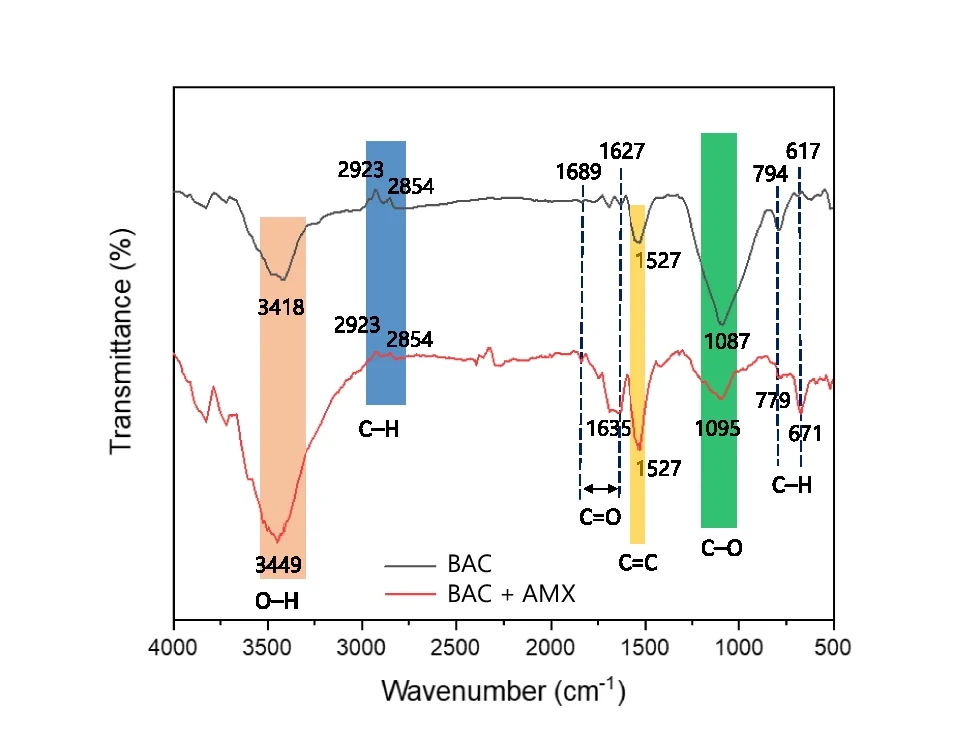
FTIR spectra of BAC before and after AMX adsorption

Figure 4 presents the FTIR spectra of pristine BAC and BAC after amoxicillin adsorption (BAC + AMX). In the spectrum of BAC, the intense band at 3418 cm^−1^ corresponds to O–H stretching vibrations. The bands at 2923 and 2854 cm^−1^ are attributed to the asymmetric and symmetric stretching vibrations of CH_2_ groups, respectively (Yusop et al. 2025b). Absorptions observed between 1700 and 1500 cm^−1^ include C = O stretching vibrations of carboxyl and carbonyl groups (1689 and 1627 cm^−1^), as well as C = C stretching of aromatic rings (1527 cm^−1^) (Martins et al. 2025; Souza et al. 2023). The band at 1087 cm⁻^1^ is assigned to C–O stretching (Laksaci et al. 2023). In addition, peaks at approximately 794 and 617 cm^−1^ are attributed to out-of-plane bending vibrations of aromatic C–H bonds, confirming the presence of aromatic structures (Belhamdi et al. 2023).

After AMX adsorption, several bands exhibited noticeable shifts (Fig. 4). The signals at 3418 cm^−1^ (O–H), 1627 cm^−1^ (C = O), 1527 cm^−1^ (C = C), and 794/617 cm^−1^ (C–H) shifted to 3449, 1635, 1535, 779, and 671 cm^−1^, respectively. These spectral changes suggest the possible involvement of hydrogen bonding during AMX adsorption. In addition, considering the aromatic structures present in both BAC and AMX, π–π interactions may also contribute to the adsorption process (Seymen et al. 2025).

The pH_PZC_ corresponds to the pH at which the adsorbent surface exhibits no net charge, as the densities of positive and negative surface charges are balanced. This parameter plays a crucial role in governing electrostatic interactions with dissolved species and, consequently, in determining adsorption behavior (Nascimento et al. 2024). The pH_PZC_ of BAC, determined from the plot of initial pH versus ΔpH (Fig. S2), was 8.65. At pH values below this point, the surface is predominantly protonated and positively charged, favoring the adsorption of anionic species through electrostatic attraction. Conversely, at pH values above the pH_PZC_, the surface becomes negatively charged, which may enhance the adsorption of cationic species.

### Stability of AMX under ultrasonic irradiation

Since ultrasonic irradiation may promote the degradation of organic compounds through cavitation-induced reactions, control experiments were performed to evaluate the stability of AMX under the sonication conditions employed in this study. AMX solutions (20 mg L^−1^) were subjected to ultrasound irradiation for 30 min in the absence of BAC, and UV–Vis spectra were recorded at 0, 10, 20, and 30 min. The resulting spectra are presented in Fig. S3. No detectable changes were observed in either the absorbance intensity or spectral profile of AMX throughout the sonication period. Furthermore, no new absorption bands were detected over the evaluated wavelength range, indicating that ultrasound irradiation did not promote measurable AMX degradation under the experimental conditions employed. Therefore, the removal of AMX observed in the subsequent experiments can be predominantly attributed to adsorption onto BAC rather than to sonochemical degradation.

### Effect of ultrasound irradiation on AMX adsorption

To evaluate the effect of ultrasound irradiation on AMX adsorption, the adsorption capacity of BAC was compared under otherwise identical experimental conditions in the absence (static conditions) and presence of ultrasound irradiation. As shown in Fig. S4, ultrasound irradiation increased the AMX adsorption capacity at all initial concentrations evaluated. At an initial AMX concentration of 20 mg L^−1^, the adsorption capacity increased from 14.72 ± 1.51 mg g^−1^ in the absence of ultrasound to 20.13 ± 2.41 mg g^−1^ under ultrasound irradiation, corresponding to an increase of approximately 36.8%. At C_0_ = 50 and 100 mg L^−1^, the adsorption capacity increased from 29.60 ± 0.78 to 35.05 ± 2.90 mg g^−1^ and from 36.30 ± 3.46 to 43.45 ± 1.63 mg g^−1^, corresponding to increases of 18.4% and 19.7%, respectively.

The enhanced adsorption observed under ultrasound irradiation may be associated with acoustic cavitation, which promotes mixing and reduces external mass-transfer limitations, thereby facilitating the transport of AMX molecules toward the accessible adsorption sites of BAC (Das and Raj 2025; Han et al. 2025). The resulting improvement in solid–liquid contact may contribute to the higher adsorption capacities observed in the ultrasound-assisted system.

### Effect of pH

**Fig. 5:**
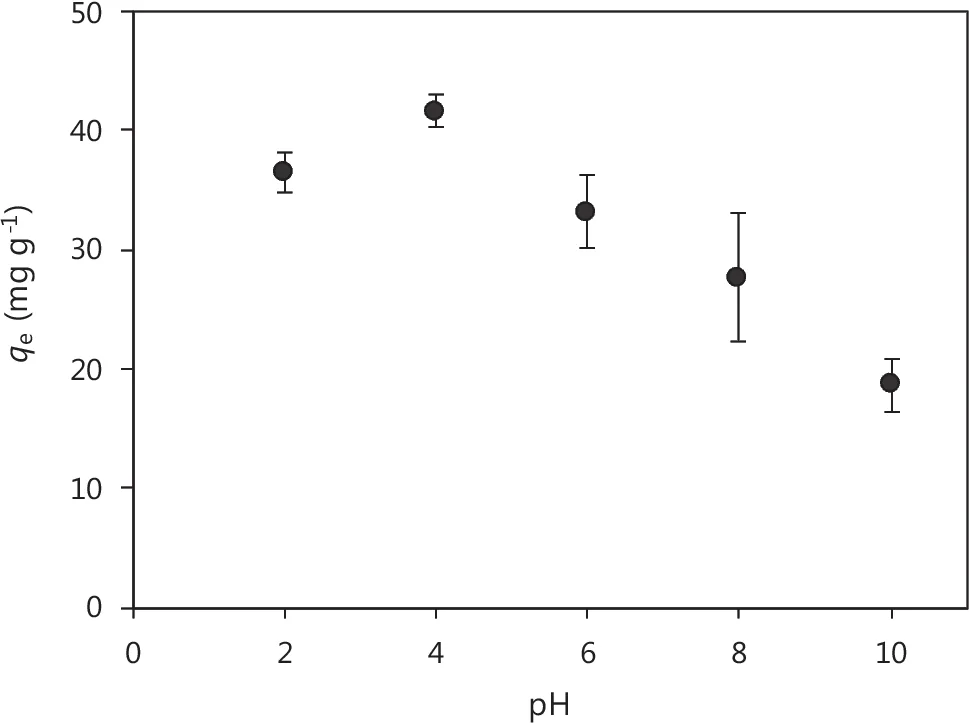
Effect of pH on the adsorption of AMX by BAC. Error bars represent the standard deviation of triplicate experiments (*n* = 3). Experimental conditions: AMX concentration = 20 mg L^−1^, adsorbent dosage = 0.2 g L^−1^, contact time: 10 min, T = 25 °C

pH is a key parameter in adsorption processes, as it controls both the ionization state of the adsorbate and the surface charge of the adsorbent, thereby influencing adsorption efficiency. To evaluate this effect, AMX solutions (20 mg L^−1^) with initial pH values ranging from 2.0 to 10.0 were treated with a fixed BAC dosage (0.2 g L^−1^). The maximum adsorption capacity was observed at pH 4.0 (41.65 mg g^−1^), followed by pH 2.0 (36.48 mg g^−1^) (Fig. 5). At higher pH values (6, 8, and 10), adsorption progressively decreased, reaching the lowest value at pH 10 (18.68 mg g^−1^).

This behavior can be interpreted considering both the pKa values of AMX functional groups (pKa_1_ = 2.4, pKa_2_ = 7.4, and pKa_3_ = 9.6; Fig. 1) and the pH_PZC_ of BAC (8.65) (Sallam et al. 2023; Shi et al. 2023). At strongly acidic pH values (pH < 2.4), AMX is predominantly present in its cationic form. At moderately acidic conditions, particularly around pH 4, zwitterionic species become more predominant, and the adsorption process is likely favored by a combination of electrostatic attraction, hydrogen bonding, and π–π interactions, resulting in the highest adsorption capacity observed in this study. As pH increases beyond pKa_2_ and pKa_3_, AMX progressively converts to anionic species (COO⁻/NH_2_/OH and COO⁻/NH_2_/O⁻). Under these conditions, electrostatic repulsion between negatively charged AMX species and the increasingly deprotonated BAC surface contributes to the decline in adsorption observed at pH 6–10. The minimum adsorption at pH 10 reflects the predominance of fully deprotonated AMX species, which interact less favorably with BAC. Comparable pH-dependent trends for AMX adsorption have been reported by Shi et al. (2023) and Haskan and Oymak (2024).

Although the highest adsorption was obtained at pH 4, subsequent experiments were conducted at the natural pH of the system (5.8), without adjustment. This decision was based on two considerations. First, operating at the natural pH provides conditions that are more representative of real environmental and industrial scenarios, where large-scale pH control is often impractical. Second, the adsorption performance at pH 5.8 was only slightly lower than that observed at pH 4, making chemical adjustment unnecessary while avoiding additional operational complexity and cost. Therefore, conducting the experiments at the natural pH enhances the practical relevance and applicability of the findings.

### Effect of BAC dosage

**Fig. 6:**
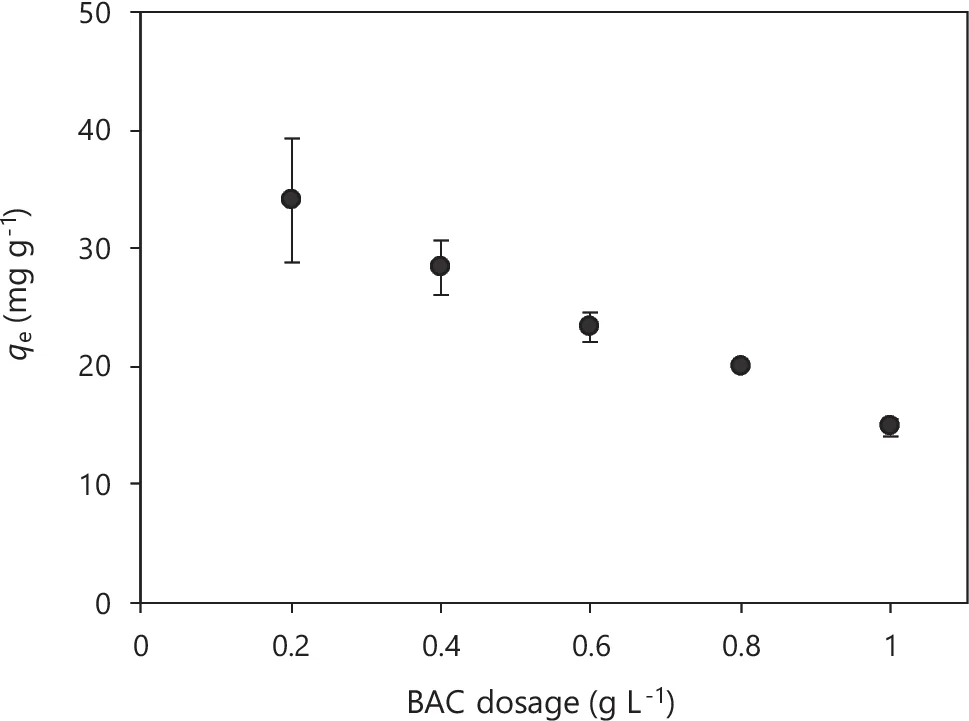
Effect of BAC dosage on AMX adsorption. Error bars represent the standard deviation of triplicate experiments (*n* = 3). Experimental conditions: AMX concentration = 20 mg L^−1^, pH = 5.8, contact time: 10, T = 25 °C

Determining an optimal adsorbent dosage is essential to avoid excessive material consumption while maintaining high removal efficiency. Therefore, the effect of BAC dosage on AMX adsorption was evaluated under the following conditions: natural pH (5.8), initial AMX concentration of 20 mg L^−1^, and 25 °C. As the BAC dosage increased from 0.2 to 1.0 g L^−1^, the adsorption capacity decreased from 34.08 to 14.85 mg g^−1^ (Fig. 6). This trend is commonly observed in batch adsorption systems and is primarily associated with the increase in adsorbent mass relative to the fixed amount of AMX available in solution, resulting in a lower adsorption capacity expressed per unit mass of adsorbent. Furthermore, at higher dosages, the adsorbate-to-adsorbent ratio decreases, leading to incomplete utilization of the available adsorption sites. In addition, particle aggregation at elevated solid concentrations may reduce the effective surface area and limit access to active sites (Santos et al. 2025).

### Effect of contact time and adsorption kinetic

Contact time is a key parameter in adsorption processes, as it directly influences both removal efficiency and operational feasibility. In practical applications, rapid contaminant uptake is highly desirable because shorter equilibrium times are associated with lower operational costs and higher treatment throughput.

**Fig. 7:**
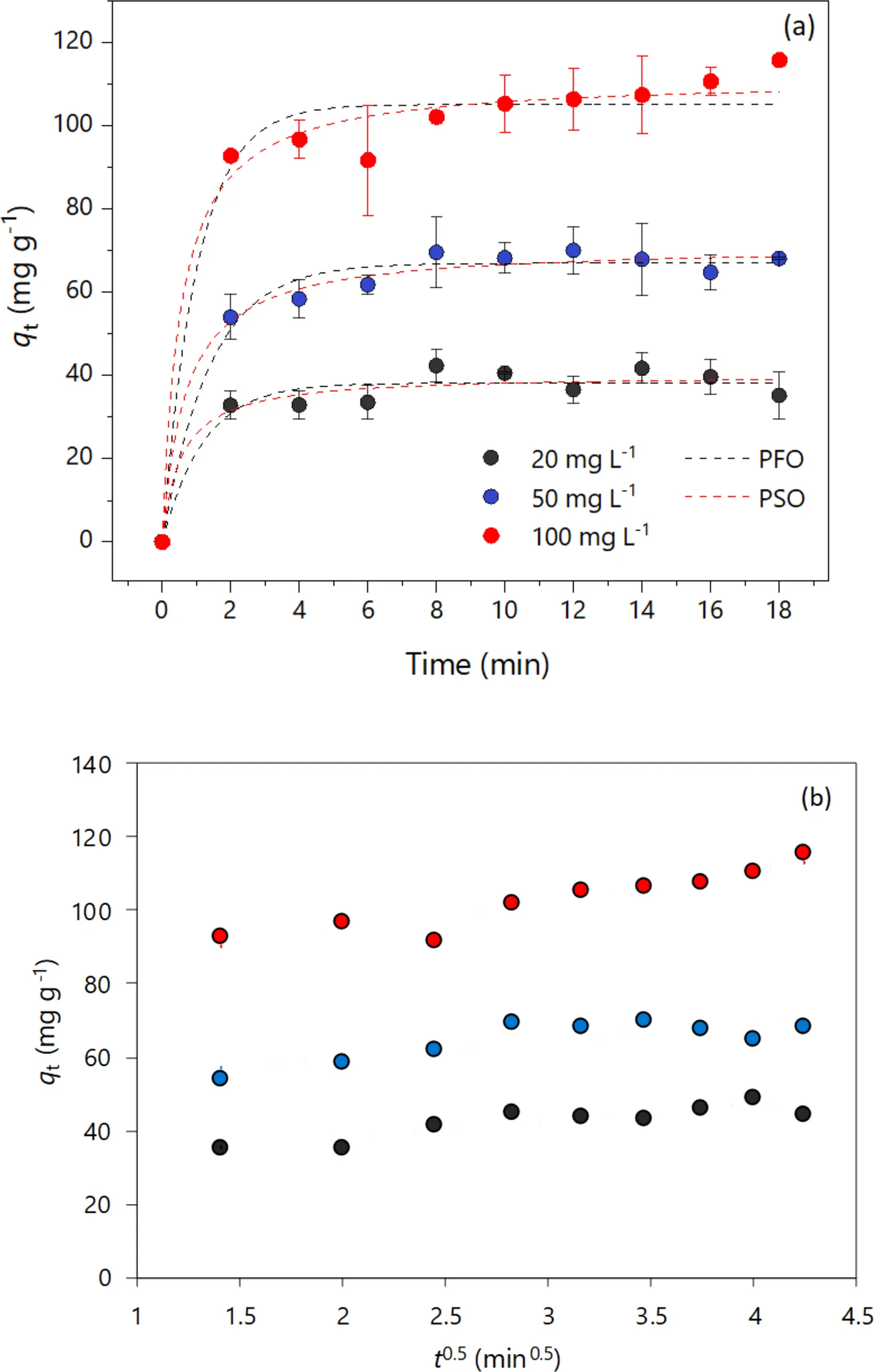
Adsorption kinetics of AMX onto BAC: PFO and PSO models (**a**), and intraparticle diffusion model (**b**). Error bars represent the standard deviation of triplicate experiments (*n* = 3). Experimental conditions: pH = 5.8, adsorbent dosage = 0.2 g L^−1^, T = 25 °C

The adsorption kinetics of AMX onto BAC were evaluated at initial concentrations of 20, 50, and 100 mg L^−1^ using an adsorbent dosage of 0.2 g L^−1^ at natural pH (5.8) and 25 °C. The kinetic profiles are presented in Fig. 7. As the initial AMX concentration increased, the adsorption capacity also increased, reflecting the greater driving force for mass transfer and the higher probability of adsorbate–adsorbent interactions at elevated solute concentrations (Sallam et al. 2023).

For all concentrations, most adsorption occurred within the first 8–10 min, followed by only slight increases until equilibrium was reached (Fig. 7). This rapid initial uptake can be attributed to the abundance of available active sites at the beginning of the process. As adsorption proceeds, these sites become progressively occupied, reducing the number of vacant sites and slowing the uptake rate until equilibrium is established (Santos et al. 2025).

In the present study, equilibrium was reached around 10 min, indicating substantially faster kinetics than many activated carbons previously reported for AMX removal. For instance, mineral-derived activated carbons required 1500–2280 min to reach equilibrium (Dolfini et al. 2024), whereas magnetic activated carbon produced from *Eucalyptus* sawdust required approximately 300 min (Shi et al. 2023). Even olive biomass-derived activated carbons, which showed comparatively faster behavior, still required 100–200 min (Rodrigues et al. 2020). The short equilibrium time observed for BAC indicates rapid adsorption kinetics and suggests favorable interactions between AMX molecules and the adsorbent surface. This characteristic is particularly attractive for practical applications, where rapid contaminant removal is essential for efficient water treatment.

The adsorption kinetics of AMX onto BAC were evaluated using three commonly applied models: pseudo-first-order (PFO), pseudo-second-order (PSO), and intraparticle diffusion. The estimated kinetic parameters are summarized in Table 1. Both the PFO and PSO models provided satisfactory fits to the experimental data (Fig. 7a). The PSO model consistently yielded slightly higher *R*^2^ values and lower root mean square error (RMSE) values than the PFO model at all investigated AMX concentrations (20, 50, and 100 mg L⁻^1^), indicating a marginally better statistical fit to the experimental data. However, this improved statistical performance should not be interpreted as evidence of a specific adsorption mechanism, since the PSO model alone cannot distinguish between physical and chemical adsorption. Instead, the adsorption kinetics are likely governed by multiple coupled transport and adsorption processes, including external film diffusion, intraparticle diffusion, and surface diffusion within the porous structure, together with the progressive occupation of adsorption sites (Işıtan 2025; Seymen et al. 2025; Yusop et al. 2025b).

**Table 1:** Estimated kinetic parameters for the adsorption of AMX onto BAC using PFO, PSO, and intraparticle diffusion models

Parameter	AMX concentration (mg L^−1^)		
	20	50	100
Experimental data (mg g^−1^)	35.16	68.05	115.78
	**Pseudo-first-order model**		
*q*_e_ (mg g^−1^)	38.20	66.99	105.12
*k*_1_ (min^−1^)	0.8312	0.7199	0.9749
*R*^2^	0.936	0.981	0.963
RMSE	3.302	3.088	6.864
	**Pseudo-second-order model**		
*q*_e_ (mg g^−1^)	40.26	71.24	111.49
*k*_2_ (mg g^−1^ min^−1^)	0.0454	0.0205	0.0167
*R*^2^	0.939	0.989	0.977
RMSE	3.045	2.485	5.109
	**Intraparticle model**		
*k*_id_ (mg g^−1^ min^−0.5^)	4.380	4.702	8.016
*C* (mg g^−1^)	29.26	50.45	78.89
*R*^2^	0.778	0.637	0.871
RMSE	2.367	3.595	3.120

To further elucidate the mass-transfer mechanism, the intraparticle diffusion model was applied. As shown in Fig. 7b, the plots of *q*_t_ versus *t*^0.5^ exhibited linear regions for all AMX concentrations but did not pass through the origin, indicating that intraparticle diffusion participated in the adsorption process but was not the sole rate-controlling step. Instead, AMX adsorption onto BAC likely involved multiple mass-transfer mechanisms. Initially, AMX molecules were transported from the bulk solution to the external surface of BAC through film diffusion. Subsequently, the coexistence of mesopores and micropores likely favored intraparticle diffusion, with mesopores serving primarily as diffusion pathways and micropores providing the main adsorption domains for AMX molecules. In addition, surface diffusion of adsorbed molecules along the internal pore walls may also have contributed to their migration toward energetically favorable adsorption sites (Stavrinou et al. 2023a, b, 2025). Therefore, the overall adsorption process likely resulted from the combined effects of external film diffusion, intraparticle diffusion through the pore network of BAC, surface diffusion along the internal pore walls, and adsorption within the microporous domains.

It should be noted that the acoustic power delivered to the liquid, power density, acoustic intensity, electrical energy consumption, and sonication efficiency were not experimentally determined in this study. Consequently, the specific contribution of ultrasonic irradiation to the mass-transfer processes described above could not be quantitatively assessed. Future studies should include experimental characterization of these parameters to enable more rigorous evaluation and comparison of ultrasound-assisted adsorption systems.

### Adsorption isotherms

**Fig. 8:**
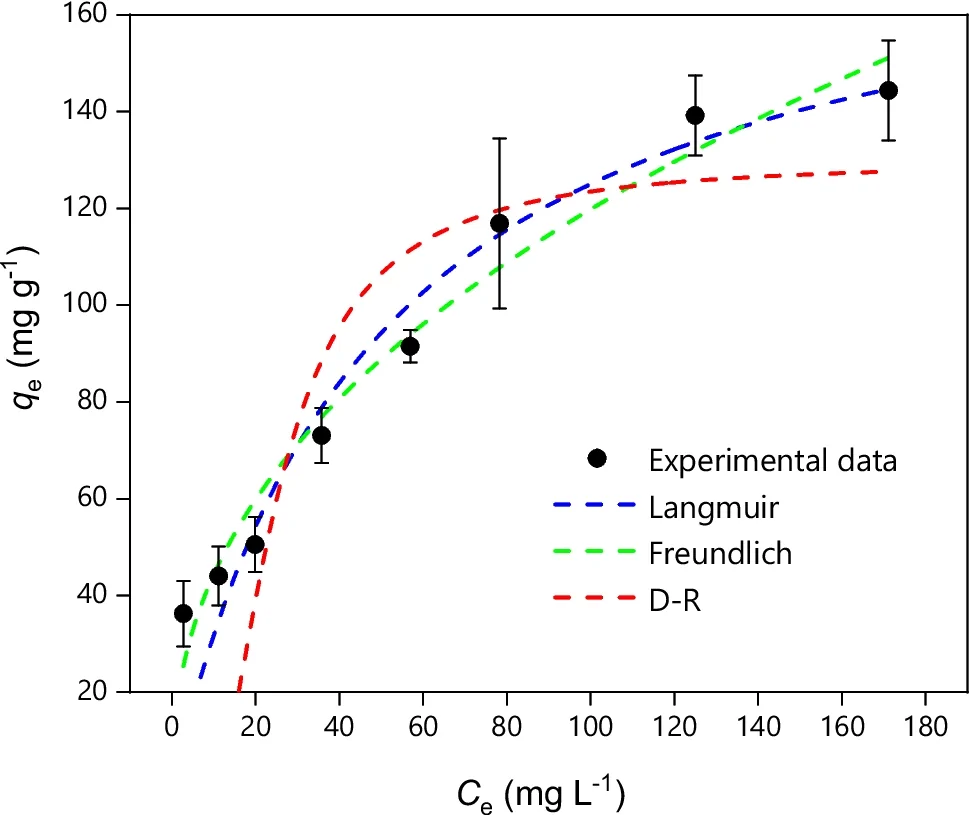
Isotherm modeling of AMX adsorption onto BAC using Langmuir, Freundlich, and D–R models. Error bars represent the standard deviation of triplicate experiments (*n* = 3). Experimental conditions: pH = 5.8, AMX concentration = 10–200 mg L^−1^, adsorbent dosage = 0.2 g L^−1^, contact time: 10 min, T = 25 °C

The adsorption equilibrium data for AMX removal by BAC were analyzed using the Langmuir, Freundlich, and Dubinin–Radushkevich (D–R) isotherm models (Fig. 8 and Table 2). Among these, the Freundlich model provided the best statistical fit, as evidenced by the highest correlation coefficient (*R*^2^ = 0.968) and the lowest root mean square error (RMSE = 7.153). The better fit of the Freundlich model suggests adsorption on a heterogeneous surface with adsorption sites exhibiting different adsorption energies, which is consistent with the empirical assumptions of the Freundlich model (Zubir et al. 2026). The Freundlich constant *n* was 2.316; since *n* > 1, the process is classified as favorable (Işıtan 2025; Yusop et al. 2025a). In addition, the Freundlich constant *K*_F_ was 16.397 (mg g^−1^) (mg L^−1^)^1/n^, indicating a considerable affinity of BAC for AMX adsorption. These findings are also consistent with the heterogeneous surface morphology observed by SEM and the coexistence of micro- and mesopores revealed by BET analysis. Similar adsorption behavior has also been reported for other adsorbents used for AMX removal, including sugarcane bagasse ash, an engineered Fe_2_O_3_–biochar composite, and chitosan–alginate composite beads, for which the Freundlich model likewise provided the best description of the equilibrium data. These studies similarly associated the better fit of the Freundlich model with the heterogeneous distribution of adsorption sites (Pinheiro et al. 2024; Saldarriaga et al. 2026; Zubir et al. 2026).

**Table 2:** Estimated parameters for Langmuir, Freundlich, and D–R models for equilibrium adsorption of AMX onto BAC

Model	Parameter	Value
Langmuir	*q*_max_ (mg g^−1^)	185.57
	*K*_L_ (L mg^−1^)	0.206
	*R*^2^	0.928
	RSME	10.789
Freundlich	n	2.316
	*K*_F_ ((mg g^−1^) (L mg^−1^)^1/n^)	16.397
	*R*^2^	0.968
	RSME	7.153
D-R	*q*_max_ (mg g^−1^)	126.87
	*K*_DR_	6.388
	*E* (kJ mol^−1^)	0.29
	*R*^2^	0.718
	RSME	188.18

The Langmuir model, which assumes monolayer adsorption on a homogeneous surface with a finite number of identical sites, showed a moderate fit (*R*^2^ = 0.928, RMSE = 10.789), inferior to that of the Freundlich model (Table 2). The maximum adsorption capacity (*q*_max_) estimated by Langmuir was 185.57 mg g^−1^, highlighting the high potential of BAC for AMX uptake. An additional parameter of the Langmuir model is the dimensionless separation factor (*R*_L_), defined as *R*_L_ = 1/(1 + *K*_L_*C*_i_), where *K*_L_ is the Langmuir constant (L mg^−1^) and *C*_i_ is the initial AMX concentration (mg L^−1^). In this study, *R*_L_ values ranged from 0.327 at 10 mg L^−1^ to 0.024 at 200 mg L^−1^. Since all values were between 0 and 1, AMX adsorption onto BAC can be classified as favorable over the investigated concentration range (Ahmadi et al. 2024). Moreover, the decrease in *R*_L_ with increasing initial concentration suggests stronger adsorption favorability at higher AMX levels.

To further assess the adsorption performance of BAC, Table 3 compares the maximum adsorption capacities (*q*_max_) reported for a wide range of adsorbents used for AMX removal, including activated carbons, biochars, nanocomposites, molecularly imprinted materials, carbon nanotube-based adsorbents, and polymeric beads. BAC exhibited one of the highest *q*_max_ values among the reported materials. However, differences in experimental conditions among studies limit direct comparison of adsorption capacities.

**Table 3:** Comparison of maximum adsorption capacities (*q*_max_) reported for AMX removal using conventional and advanced adsorbent materials

Adsorbent	*q*_max_ (mg g^−1^)	References
Magnetite iron nanoparticles	6.47	Ahmadi et al. (2024)
Calcium-rich biochars	14.6–46.8–56.2	Jellali et al. (2024)
Magnetic biochar from pineapple peel	18.6	Haskan and Oymak (2024)
Activated carbon from termite bio-waste	23.4	Niero et al. (2020)
Activated carbon from murici seeds	28.3	Martins et al. (2025)
Activated carbon from *Aloe vera* leaf waste	28.86–29.11	Hashemzadeh et al. (2022)
Molecularly imprinted hollow microspheres	57.6	Li et al. (2021)
Ag_2_O-CuO/biochar	64.94	Allwar et al. (2023)
Jujube-derived activated carbons	77.79	Belaissa et al. (2022)
Bismuth oxyiodide–chitosan nanocomposite	81.01	Bisaria et al. (2022)
TiO_2_/nano-hidroxiapatita	88.46	Alshandoudi et al. (2023)
Silica nanoparticles from rice husks	99.8	Elnazer et al. (2024)
Multi-Walled Carbon Nanotubes functionalized with glycine and polypyrrole	102.09	Khazaie et al. 2025
Chitosan beads	102.2	Pinheiro et al. (2024)
Alginate beads	104.3	Pinheiro et al. (2024)
Magnetite/chitosan/*Chlorella vulgaris* composite	140.2	Mirizadeh et al. (2024)
Magnetite/chitosan/*Arthrospira platenses* composite	150.8	Mirizadeh et al. (2024)
Graphene–Aminated Lignin composite	156.25	Bhuiyan et al. (2026)
Coffee grounds biochar	178.57	Choi et al. (2023)
**BAC**	**185.57**	**This work**
Oil palm trunk derived activated carbon	238.67	Yusop et al. (2025a)

The D–R model provided a poorer fit to the experimental equilibrium data than the Freundlich and Langmuir models (*R*^2^ = 0.718) (Table 2). Therefore, the mean adsorption energy (*E*) derived from this model was not used to infer the adsorption mechanism, since the D–R model did not adequately describe the experimental equilibrium data.

### Thermodynamic studies

The thermodynamic parameters for AMX adsorption onto BAC were evaluated to better understand the feasibility and energetic nature of the process. The standard Gibbs free energy change (ΔG) was negative over the entire temperature range studied, varying from –1.017 kJ mol⁻^1^ at 10 °C to –3.200 kJ mol⁻^1^ at 50 °C (Table 4), confirming the spontaneous nature of the adsorption process. The increasingly negative ΔG values with rising temperature indicate that spontaneity was enhanced at higher temperatures.

**Table 4:** Thermodynamic parameters for adsorption of AMX onto BAC

*T* (ºC)	*K*_D_	Δ*G* (kJ mol^−1^)	Δ*H* (kJ mol^−1^)	Δ*S* (J mol^−1^ K^−1^)
10	1.54	− 1.017	12.52	48.6
25	2.51	− 2.290		
40	2.98	− 2.850		
50	3.23	− 3.200		

The positive enthalpy change (ΔH = 12.52 kJ mol⁻^1^) and entropy change (ΔS = 48.6 J mol⁻^1^ K⁻^1^), obtained from the slope and intercept of the Van’t Hoff equation (Fig. S5), indicate that the adsorption process was endothermic and accompanied by increased randomness at the solid–liquid interface. This increase in entropy may be associated with desolvation effects and rearrangement of water molecules surrounding both the adsorbate and adsorbent (Belhamdi et al. 2023).

### Performance of machine learning algorithms

Tree-based machine learning algorithms were employed to model the adsorption capacity of AMX onto BAC and evaluate the influence of operational variables on process performance. The predictive performance of the models was assessed using the coefficient of determination (*R*^2^), root mean square error (RMSE), and mean absolute error (MAE) for both training and test datasets. The results are summarized in Table 5.

**Table 5:** Predictive performance of Decision Tree, Random Forest, and Extra Tree models for AMX adsorption capacity prediction

Model	Dataset	*R*^2^	RMSE	MAE
Decision Tree	Training	0.999	1.241	0.331
	Test	0.938	8.692	6.330
Random Forest	Training	0.989	3.402	2.716
	Test	0.964	6.580	4.915
Extra Tree	Training	0.999	1.241	0.331
	Test	0.983	5.573	3.926

The DT model achieved a high coefficient of determination during the training phase (*R*^2^ = 0.999), accompanied by very low RMSE and MAE values. However, its predictive performance decreased in the test dataset (*R*^2^ = 0.938), indicating a certain degree of overfitting (Table 5). This behavior is commonly observed in single decision-tree models, which tend to capture noise and specific patterns from the training data, thereby reducing their generalization capability when applied to unseen datasets (Amro et al. 2021).

In contrast, the ensemble-based algorithms exhibited improved predictive performance on the evaluated datasets. The RF model achieved high predictive accuracy for both datasets, with *R*^2^ values of 0.989 and 0.964 for the training and test sets, respectively (Table 5). The use of multiple decision trees generated from bootstrapped datasets reduces model variance and enhances robustness, resulting in more reliable predictions (Ghaedi et al. 2014).

Among the evaluated algorithms, the ET model exhibited the best predictive performance. As shown in Table 5, this model achieved the highest test accuracy (*R*^2^ = 0.983) and the lowest prediction errors (RMSE = 5.573 and MAE = 3.926). The higher predictive performance of the ET algorithm in the present dataset may be related to the higher degree of randomization employed during tree construction, although further validation with larger datasets would be required to confirm its generalization to independent datasets (Zhang et al. 2026).

**Fig. 9:**
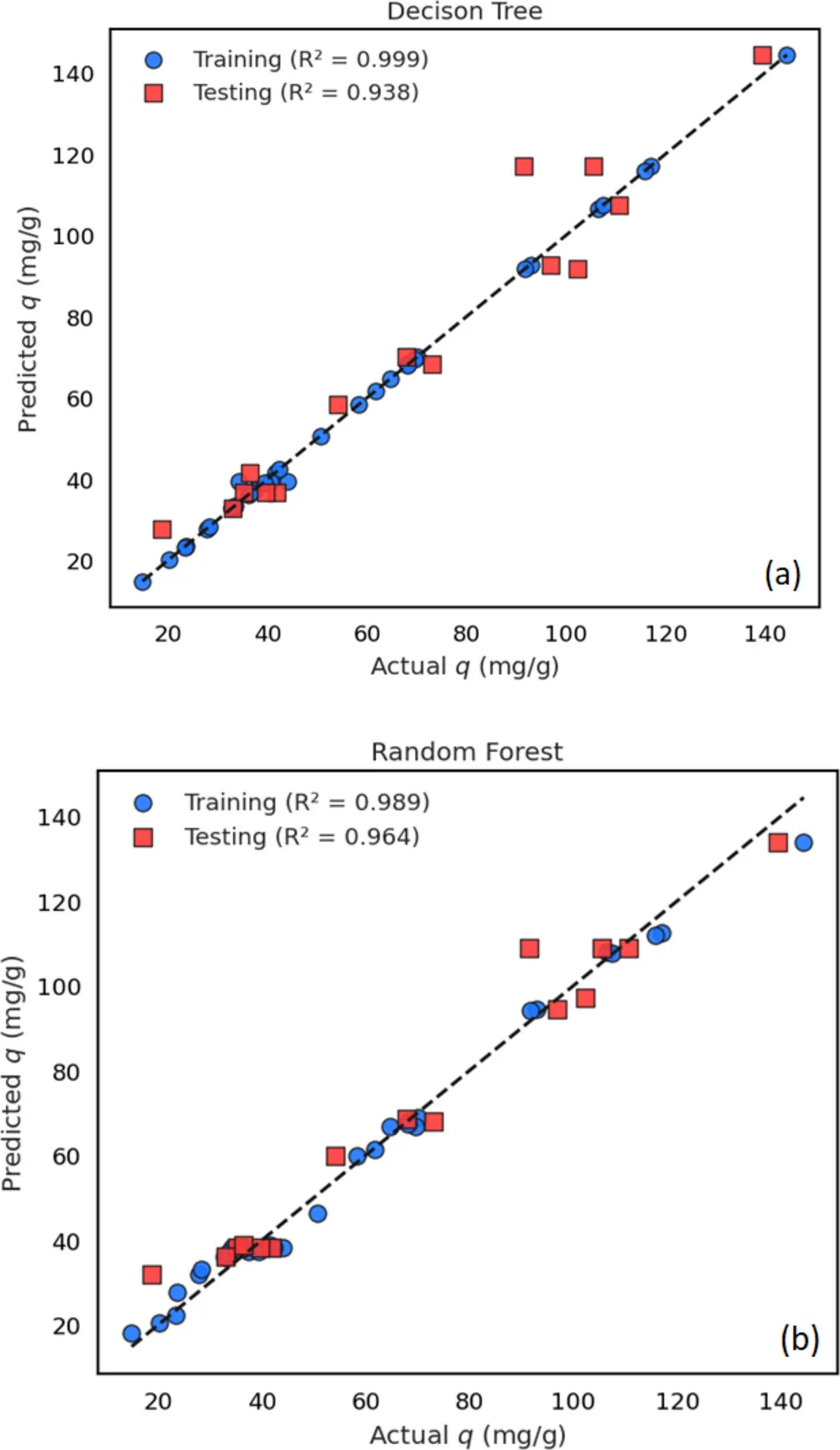

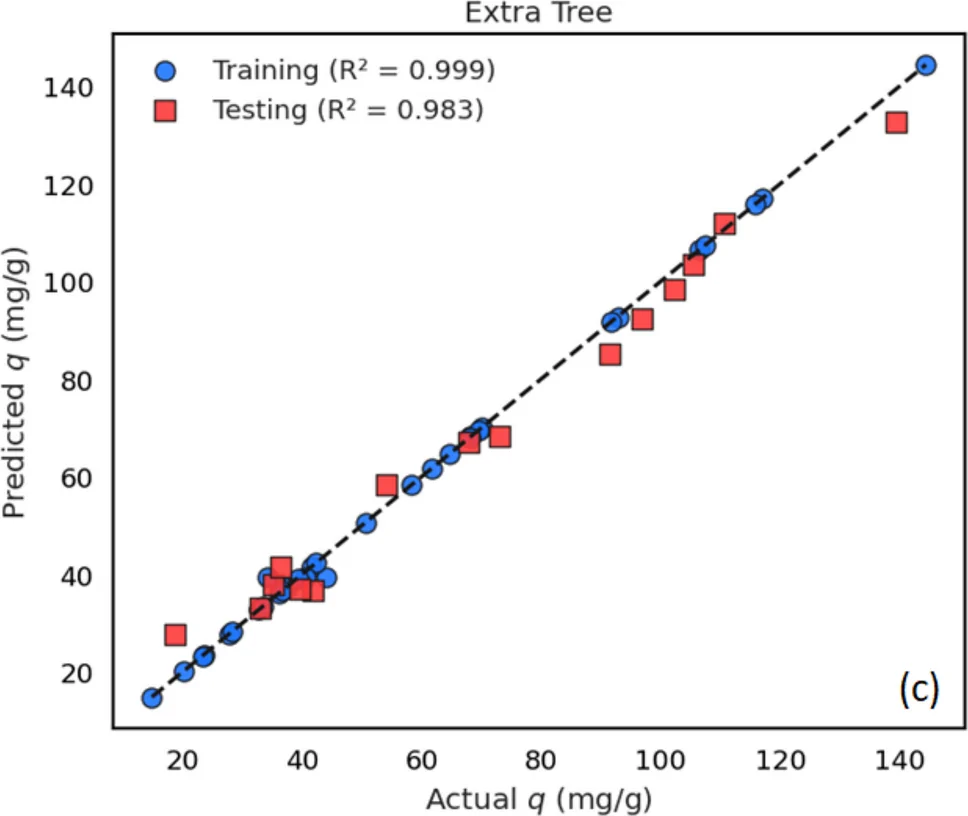
Experimental versus predicted adsorption capacities obtained using Decision Tree (**a**), Random Forest (**b**), and Extra Tree (**c**) models for the training and test datasets

The predictive capability and generalization behavior of the models are further illustrated in Fig. 9, which compares the experimental and predicted adsorption capacities for the training and test datasets. The DT model (Fig. 9a) shows greater dispersion of test data around the 1:1 line, indicating larger prediction errors and lower generalization capability. In contrast, the RF model (Fig. 9b) exhibited improved agreement between experimental and predicted values, with reduced residual dispersion. Among the evaluated models, the ET algorithm (Fig. 9c) demonstrated the best predictive performance, with most points closely distributed along the 1:1 line, indicating its better predictive performance for the present dataset.

The successful application of tree-based machine learning algorithms in this study is consistent with recent reports demonstrating the effectiveness of ensemble-learning approaches for modeling adsorption processes involving emerging contaminants. Seymen et al. (2025), for example, applied Decision Tree, Random Forest, and Extreme Gradient Boosting models to predict AMX removal using biomass-derived activated carbons and obtained high predictive accuracy. Similarly, Zhang et al. (2023) showed that Random Forest models could effectively predict tetracycline adsorption onto biochar by capturing nonlinear relationships between adsorbent properties and operational conditions. More recently, Pei et al. (2026) developed machine learning models to predict the maximum adsorption capacity of antibiotics onto biochar using a curated adsorption dataset. Among the evaluated algorithms, Extreme Gradient Boosting achieved the highest predictive performance, while Random Forest also demonstrated strong predictive capability. These findings reinforce the suitability of ensemble tree-based algorithms for modeling complex adsorption systems.

In addition to predictive performance, the relative importance of the operational variables was evaluated using tree-based machine learning models (Fig. S6). For all evaluated algorithms, the initial AMX concentration (*C*_i_) was identified as the most influential variable, with importance values of 0.948, 0.949, and 0.931 for the DT, RF, and ET models, respectively. This finding indicates that the concentration gradient between the solution and the adsorbent surface was the main driving force governing adsorption capacity.

Contact time and adsorbent dosage showed secondary contributions to the predictive models, although their relative importance varied slightly among the algorithms. In the ET model, adsorbent dosage was identified as slightly more important than contact time, whereas the opposite trend was observed for the DT and RF models, in which contact time showed slightly greater importance than adsorbent dosage. In contrast, temperature and pH exhibited the lowest importance values across all models, indicating a comparatively smaller influence on adsorption performance within the investigated experimental conditions.

Despite the satisfactory predictive performance obtained, the machine-learning analysis should be regarded as exploratory because the dataset comprised only 48 experimental observations and model evaluation was based on a single 70/30 training–test partition. Although five-fold cross-validation was used during hyperparameter optimization, this procedure does not fully account for the variability associated with alternative data partitions. Therefore, the reported predictive performance should not be interpreted as definitive evidence of model generalizability. Future studies based on larger experimental datasets and repeated or nested cross-validation are recommended to provide more robust estimates of model performance.

### Regeneration and reuse performance of BAC

**Fig. 10:**
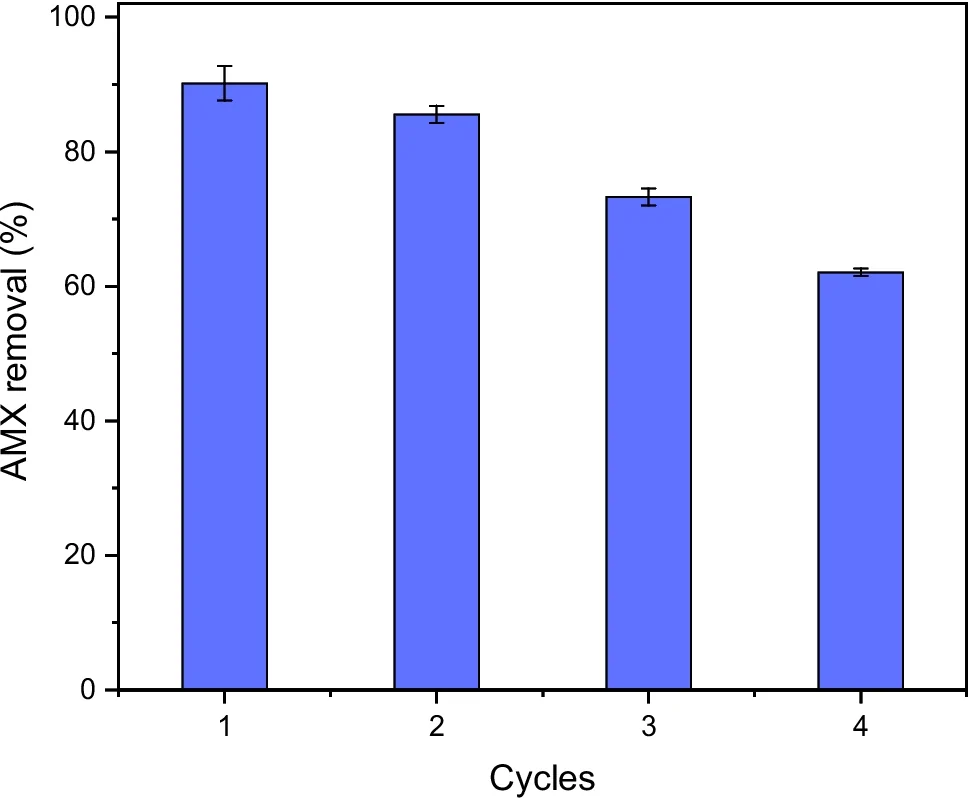
Regeneration and reuse performance of BAC for AMX adsorption over successive adsorption–desorption cycles

The regeneration and reusability of BAC were evaluated over four consecutive adsorption–desorption cycles to assess its potential for repeated use in AMX removal. After each adsorption cycle, the exhausted adsorbent was regenerated using NaOH solution to promote AMX desorption. The removal efficiencies obtained during the successive cycles are presented in Fig. 10.

BAC exhibited high adsorption performance during the first cycle, achieving an AMX removal efficiency of 90.2%. After regeneration, the adsorbent maintained relatively high efficiencies in the subsequent cycles, with removal values of 85.6, 73.3, and 62.1% for the second, third, and fourth cycles, respectively. Although a progressive decline in adsorption performance was observed, BAC still retained a considerable fraction of its adsorption capacity after four reuse cycles. The gradual reduction in removal efficiency may be attributed to incomplete desorption of AMX molecules from the porous structure, resulting in the partial blockage of pore channels and active adsorption sites (Chakinala and Gogate 2024). Despite these promising results, additional studies involving a larger number of regeneration cycles are necessary to assess the long-term stability and regeneration performance of BAC.

### Proposed adsorption mechanism

**Fig. 11:**
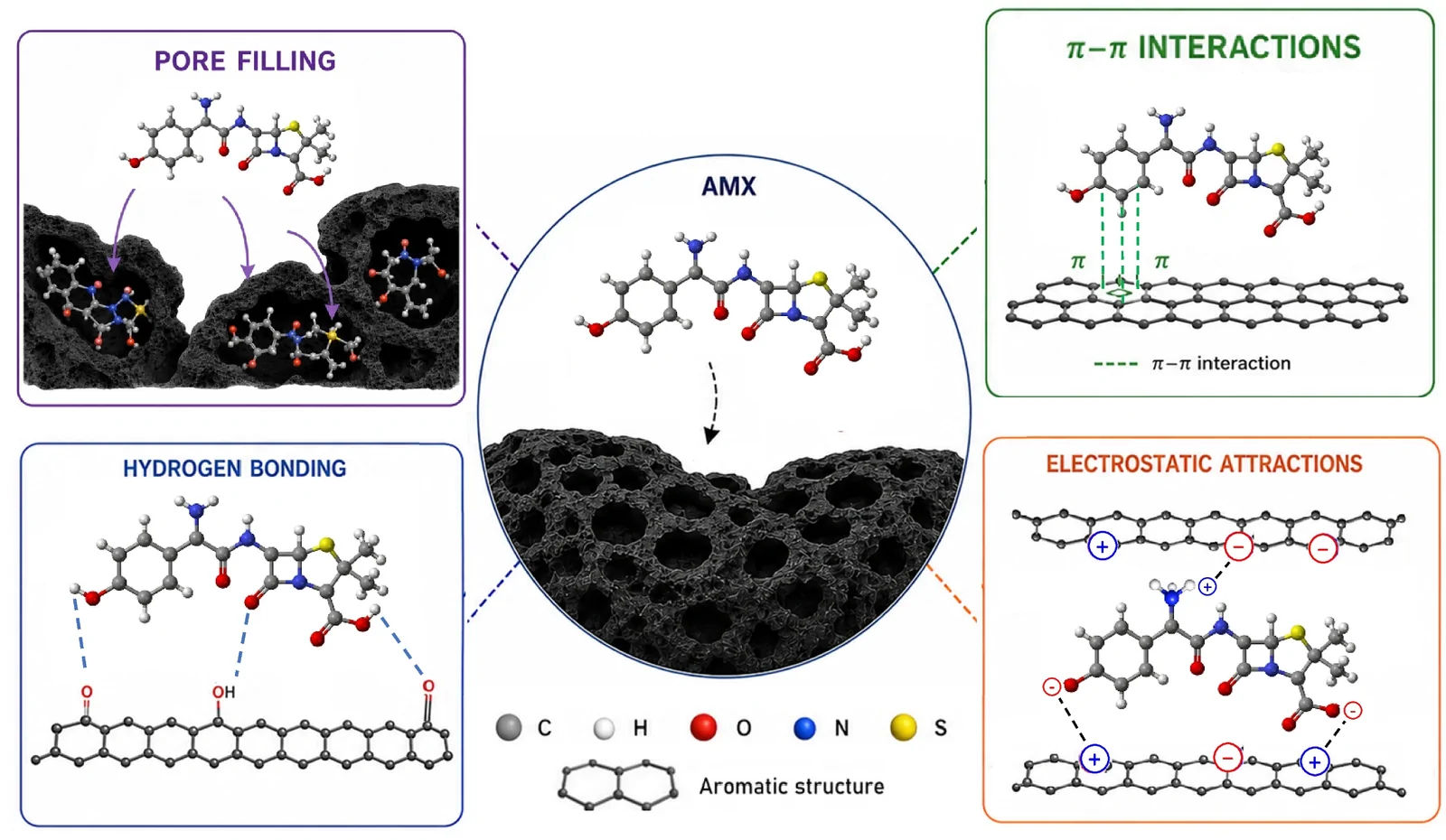
Proposed adsorption mechanism of AMX onto BAC

Based on the physicochemical characterization and adsorption behavior, a plausible adsorption mechanism is proposed for the uptake of AMX onto BAC, involving pore filling, hydrogen bonding, π–π interactions, and electrostatic attractions (Fig. 11). The coexistence of micro- and mesopores, as revealed by BET analysis, suggests that pore filling may have contributed to the diffusion and accommodation of AMX molecules within the porous BAC structure. The shifts observed in the FTIR vibrational bands are consistent with the possible involvement of hydrogen bonding between functional groups of the antibiotic and oxygen-containing functional groups on the adsorbent surface. Furthermore, π–π stacking interactions may also occur between the aromatic ring of AMX and the graphitic domains of the carbon matrix. The influence of solution pH on adsorption capacity further suggests that electrostatic interactions may also have contributed to the adsorption process, depending on the ionization state of AMX and the surface charge of BAC. Similar adsorption mechanisms have been proposed for other activated carbons used for AMX removal (Shi et al. 2023; Yusop et al. 2025a, b).

## Conclusion

This study demonstrated that babassu-derived activated carbon (BAC) is a promising adsorbent for amoxicillin removal under ultrasonic irradiation. Adsorption equilibrium was achieved within approximately 10 min, indicating rapid adsorption kinetics for AMX removal. Maximum adsorption was observed under mildly acidic conditions (pH 4). Kinetic data were better described by the pseudo-second-order model, while the Freundlich isotherm provided the best fit to the equilibrium data, suggesting adsorption on a heterogeneous surface. BAC also exhibited a high adsorption capacity (185.57 mg g^−1^), which is within the upper range of values reported for various adsorbents used for AMX removal. Thermodynamic analysis confirmed that the adsorption process was spontaneous and endothermic, while regeneration experiments demonstrated satisfactory reusability of the material over successive cycles. Tree-based machine-learning algorithms provided satisfactory predictive performance for the present dataset, with the Extra Trees model showing the best performance among the evaluated algorithms. Feature importance analysis identified the initial AMX concentration as the most influential variable within the evaluated dataset. These findings highlight the adsorption performance of BAC under ultrasonic irradiation and the potential of tree-based machine-learning models for adsorption prediction. While the present study demonstrated the promising potential of BAC for AMX removal under controlled laboratory conditions, further investigations using real wastewater are necessary to assess its practical applicability. The influence of complex water matrices, including ionic strength, natural organic matter, and competing ions, should be evaluated to support future scale-up and environmental applications. In addition, future studies should include experimental characterization of the acoustic power delivered to the liquid, power density, acoustic intensity, energy consumption, and sonication efficiency to enable a quantitative assessment of the contribution of ultrasound to adsorption and facilitate comparisons and scale-up of ultrasonic systems.

## Supplementary Information

Below is the link to the electronic supplementary material.

ESM 1 (DOCX 422 KB)

## Data Availability

The datasets generated and/or analyzed during the current study will be made available from the corresponding author on reasonable request.

## Declarations

### Declaration of AI-assisted technologies in the writing process

During the preparation of this work, the authors used ChatGPT (GPT-5, OpenAI) to assist with language editing and improving the clarity and readability of the manuscript. After using this tool, the authors carefully reviewed and edited the content as needed and take full responsibility for the final content of the publication.
