# Supplementary material for: Amoxicillin removal by babassu-derived activated carbon under ultrasonic irradiation: adsorption performance and tree-based machine learning modeling
Source: Environ Sci Pollut Res Int. 2026 Oct 29;33(40):18059–76. doi: 10.1007/s11356-026-38361-z (PMC13769761; doi:10.1007/s11356-026-38361-z)
Supplement: Supplementary file 1 [file 11356_2026_38361_MOESM1_ESM.docx]

**S****upplementary materials**

**Ultrafast ultrasound-assisted amoxicillin removal using babassu-derived activated carbon: adsorption performance, regeneration, and tree-based machine learning modeling**

Luiz Eduardo Carvalho Medeiros^a^, Rossean Golin^b^, Jéssica Aparecida Ribeiro Ambrosio^c^, Andreza Ribeiro Simioni^c^, Amanda Alcaide Francisco Fukumoto^a,b^, Eduardo Beraldo de Morais^a,b*^

^a^ Postgraduate Program in Water Resources, Federal University of Mato Grosso, Cuiabá, MT, Brazil.

^b^ Department of Sanitary and Environmental Engineering, Federal University of Mato Grosso, Cuiabá, MT, Brazil.

^c^ Research and Development Institute, Vale do Paraíba University, São José dos Campos, SP, Brazil

* Corresponding author. E-mail: eduardo.morais@ufmt.br

**Figure S1.** Schematic diagram of the ultrasonic probe system used for AMX adsorption.

**Figure S2.** Point of zero charge (pH_PZC_) of BAC.

**Figure S3.** UV–Vis spectra of AMX solution (20 mg L^-1^) subjected to ultrasonic irradiation for 0, 10, 20 and 30 min in the absence of activated carbon.

**Figure S4.** Comparison of AMX adsorption capacity in the absence and presence of ultrasound irradiation at different initial concentrations (C₀): (a) 20 mg L^-1^, (b) 50 mg L^-1^, and (c) 100 mg L^-1^. Bars represent the mean ± standard deviation (n =3). Experimental conditions: adsorbent dosage = 0.2 g L^-1^, pH = 5.8, contact time: 10 minutes, T = 25 °C.

**Figure S5.** Van’t Hoff plot (ln*K*_D_ versus 1/*T*) for the adsorption of AMX onto BAC.

**Figure S6.** Relative importance of the operational variables (*C*i, contact time, adsorbent dosage, temperature, and pH) determined by the Decision Tree (a), Random Forest (b), and Extra Trees (c) models for AMX adsorption onto BAC.

**Table S1.** Mathematical expressions and parameters of the kinetic and isotherm models used for AMX adsorption.

| Model | Equation | Reference |
| --- | --- | --- |
| *Kinetic models* |  |  |
| Pseudo-first order | $q_{t}= q_{e}(1-e^{{-K}_{1}t}$) | Lagergren (1898) |
| Pseudo-second order | $q_{t}=\frac{K_{2}{q_{e}}^{2}t}{1+K_{2}q_{e}t}$ | Ho; Mckay (1999) |
| *Isotherms models* |  |  |
| Langmuir | $q_{e}= \frac{q_{\max}K_{L}C_{e}}{1+K_{L}C_{e}}$ | Langmuir (1918) |
| Freundlich | $q_{e}= K_{F}{C_{e}}^{1/n}$ | Freundlich (1906) |
| Dubinin-Radushkevich (D-R) | $q_{e}= q_{\max}e^{-\beta\varepsilon^{2}}$ | Dubinin; Radushkevich (1947) |
|  | $\varepsilon=RT ln\left( 1+ \frac{1}{C_{e}} \right)$ |  |

*q_e_* (mg g^-1^): adsorption capacity at equilibrium

*q_t_* (mg g^-1^): adsorption capacity at time *t*

*K*_1_ (min^-1^): pseudo-first-order rate constant

*K*_2_ (g mg^-1^ min^-1^): pseudo-second-order rate constant

*q_max_* (mg g^-1^): maximum adsorption capacity

*C_e_* (mg L^-1^): adsorbate concentration in solution at equilibrium

*K_L_* (L mg^-1^): Langmuir constant

*K_F_* ((mg g^-1^) (L mg^-1^)^1/n^): Freundlich constant

*n*: heterogeneity factor

*ɛ*: Polanyi potential

*β* (mol^2^ kJ^−2^): adsorption energy constant

*R*: universal gas constant (8.314 J mol^-1^ K^-1^)

**Table S2.** Mathematical equations employed in the thermodynamic analysis.

| Thermodynamic equations | Parameter |
| --- | --- |
|  |  |
| $\Delta G= -RT \ln K_{D}$  $\ln K_{D}= \frac{\Delta S}{R}- \frac{\Delta H}{R}x\frac{1}{T}$ | Δ*G*: Gibbs Free Energy Change  Δ*H*: Enthalpy Change  Δ*S*: Entropy Change *K_D_* (*qe*/*Ce*): distribution coefficient of adsorption  *T*: temperature (K)  *R*: universal gas constant (8.314 J K^-1^ mol^-1^) |
|  |  |

**Table S3.** Hyperparameter search space explored during GridSearchCV and optimal hyperparameter values for the machine learning models.

| **Model** | **Hyperparameter** | **Search space** | **Optimal value** |
| --- | --- | --- | --- |
| **Decision Tree (DT)** | max_depth | None, 3, 5, 7, 10 | None |
|  | max_features | None, sqrt, log2 | None |
|  | min_samples_leaf | 1, 2, 4 | 1 |
|  | min_samples_split | 2, 5, 10 | 2 |
| **Random Forest (RF)** | n_estimators | 50, 100, 200 | 100 |
|  | max_depth | None, 5, 10, 20 | 5 |
|  | max_features | None, sqrt, log2 | None |
|  | min_samples_leaf | 1, 2, 4 | 1 |
|  | min_samples_split | 2, 5, 10 | 2 |
| **Extra Trees (ET)** | n_estimators | 50, 100, 200 | 100 |
|  | max_depth | None, 5, 10, 20 | None |
|  | max_features | None, sqrt, log2 | None |
|  | min_samples_leaf | 1, 2, 4 | 1 |
|  | min_samples_split | 2, 5, 10 | 2 |

**References**

Dubinin MM, Radushkevich LV (1947) Equation of the characteristic curve of activated charcoal. Proc USSR Acad Sci 55:331–333.

Freundlich H (1906) Über die adsorption in Lösungen. Z Phys Chem 57:385–470.

Ho Y, McKay G (1999) Pseudo-second order model for sorption processes. Process Biochem 34(5):451–465. <https://doi.org/10.1016/S0032-9592(98)00112-5>

Lagergren SY (1898) Zur theorie der sogenannten adsorption gelöster stoffe. Kungliga Svenska Vetenskapsakad Handl 24:1–39.

Langmuir I (1918) The adsorption of gases on plane surfaces of glass, mica and platinum. J Am Chem Soc 40(9):1361–1403.
